# Feelin’ it: Differential oxidative stress sensing mediated by Cyclin C

**DOI:** 10.15698/mic2015.09.228

**Published:** 2015-09-07

**Authors:** W. Scott Moye-Rowley

**Affiliations:** 1 Department of Molecular Physiology and Biophysics, Carver College of Medicine, University of Iowa, Iowa City, IA 52242 USA.

**Keywords:** cyclin, oxidative stress, subcellular localization, stress sensing

Microbial cells that live exposed directly to their environmental milieu are faced with the challenge of adapting to the dynamic stress conditions that will inevitably be encountered. These stress conditions may vary over wide ranges and the most efficient responses would be tuned to produce a proportional buffering change. A mild stress would most efficiently be dealt with by a mild metabolic reprogramming that would prevent serious damage. A more severe environmental challenge would demand a more dramatic cellular compensatory response.

This notion of tuning cellular replies to environmental stresses lies at the heart of the manuscript from the Cooper laboratory published in this issue of *Microbial Cell* 1. Her group has been studying the surprisingly complex regulation of the transcriptional Mediator component Cyclin C 2. The Cyclin C protein in *Saccharomyces cerevisiae* is encoded by the *CNC1* gene (protein designated Cnc1) and is the cyclin for the Cdk8 cyclin-dependent protein kinase encoded by the *CDK8* gene. Cdk8 and Cnc1 work together with the Mediator proteins Med12 and Med13 to function as a multiprotein transcriptional regulatory complex acting to modulate function of the core Mediator complex (recently reviewed in 3). Cnc1:Cdk8 typically acts to repress gene expression, notably of genes involved in the stress response or meiotic program.

**Figure 1:**
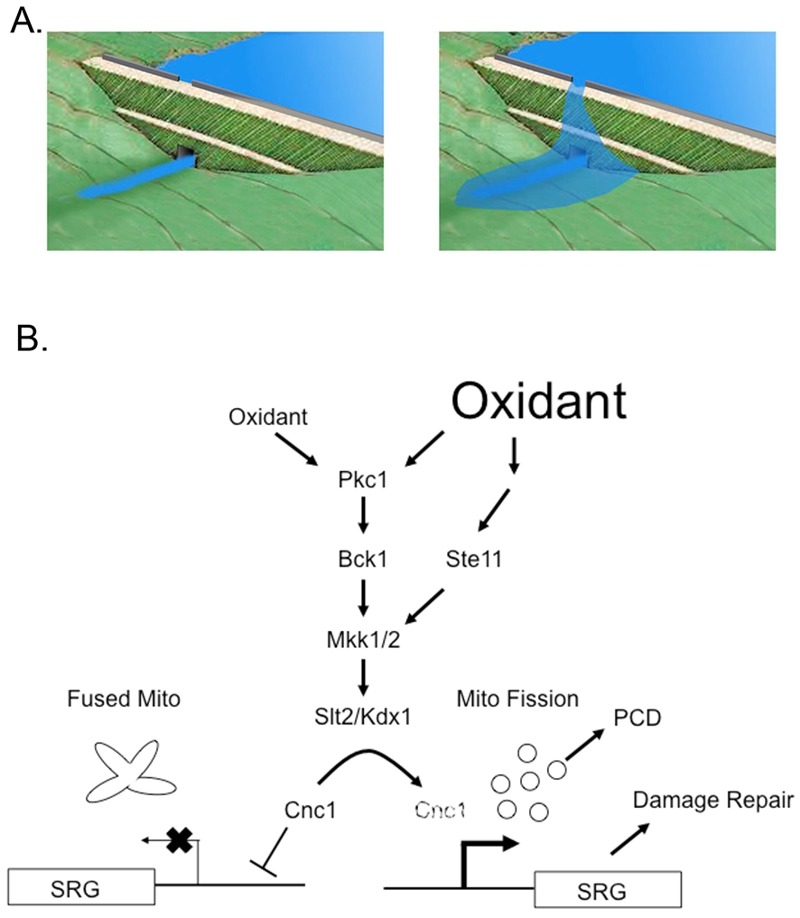
FIGURE 1: Differential signaling during oxidative stress via the cell wall integrity (CWI) pathway. **(A)** The top left panel shows a dam with a spillway at the top. Low water levels are retained behind the dam and the only outflow is via the dam outlet at its base. The top right panel shows the result when water levels crest the dam and flow over the spillway. This leads to a larger downstream output. **(B)** Signaling under either low (left side) or high (right side) oxidative stress conditions through the CWI pathway is shown. Note that these signaling events lead to the eventual degradation of cyclin C (Cnc1) as indicated by the dashed text. When Cnc1 is present, stress-responsive genes (SRG) are repressed and mitochondria exhibit a multilobe, fused morphology. Activation of the CWI pathway triggers Cnc1 degradation with accompanying mitochondrial fission, SRG activation and either programmed cell death (PCD) or repair of the stress-induced damage. See the text for further details.

In this most recent study 1, new insight is provided on how cells can distinguish between a relatively mild (0.4 mM) or more serious (1.2 mM) oxidative stress trigged by exposure to H_2_O_2_. Previous work established that a variety of stresses, including oxidants, trigger the relocalization of Cnc1 from its usual nuclear residence to the mitochondria. This localization is followed by degradation of Cnc1 but only after the protein has induced mitochondrial fission and, in many cases, programmed cell death 4. Analyses of the signaling pathway regulating Cnc1 turnover demonstrated that the cell wall integrity (CWI) pathway was required for this mode of regulation. The CWI pathway 5 is composed of the *S. cerevisiae* protein kinase C homologue (Pkc1) that acts upstream of the MAP Kinase (MAPK) module consisting of the MAPKKK Bck1, the MAPKK Mkk1 and Mkk2 (referred to as Mkk1/2) and the MAPK Slt2 (Figure 1). Previous experiments indicated that under conditions of relatively mild oxidative stress (0.4 mM H_2_O_2_) function of the MAPKKK Bck1 is required to induce degradation of Cnc1. Here, these authors discover that challenging cells with a more severe oxidative stress (1.2 mM H_2_O_2_), Cnc1 degradation occurs in a Bck1 independent manner. Further experiments revealed that a similar MAPK module, involved in mating and cell type specification, was also capable of triggering high oxidative stress-inducible degradation of Cnc1. Ste11, the MAPKKK of this parallel pathway, was already known to function in parallel with the CWI pathway 6.

Data provided in this work provide illumination of the linkage between the CWI pathway and Ste11. Earlier findings determined that the CWI pathway sensor protein Mtl1 was required for normal H_2_O_2_ inducible gene expression 7. Importantly, this defective gene expression program could be restored by introduction of a genetically activated form of the small GTPase Rho1 but not a hyperactive allele of *BCK1*. Rho1 is known to act upstream of Pkc1, among other effectors 8. Using these findings as a starting point, Cooper and colleagues showed that constitutively active Rho1 signals through the CWI pathway in a Bck1 independent manner but only when cells are challenged with a strong oxidative stress. Rho1 was found to signal to Pkc1 and then on to Ste11. This high oxidative stress-specific pathway appears to provide an additional input controlling Cnc1 degradation although Ste11 and Bck1 are both required for the high efficiency export of Cnc1 from the nucleus to the cytoplasm.

Dissection of the signaling by Ste11 supports the view that this kinase acts upstream of the MAPKKs (Mkk1/2) of the CWI pathway to stimulate Cnc1 degradation during severe oxidative stress. In response to this strong stress signal, cross-pathway signaling is activated. As pointed out by these authors, this provides a mechanism for understanding how cells can discriminate between mild and strong inputs from the environment. As indicated by the cartoon in figure 1, this differential stress sensing resembles the behavior of spillway over a dam. When the water level (or stress) is low, the dam can regulate the flow of water without invoking a second water release system (spillway). However, when water levels exceed the capacity of the dam to regulate water flow, then water flows over the spillway, leading to a stronger downstream output. In this simple model, the magnitude of the input signal can regulate the stronger downstream output. The linear activation of the CWI pathway acts as the initial limit for the downstream response but a stronger oxidative stress is capable triggering cross-pathway signaling through the Ste11 MAPKK.

The role of Ste11 in regulation of Cnc1 turnover is an important step forward in understanding the integration of stress regulation of gene expression but raises a number of new questions. Detailed analyses of the subcellular trafficking of Cnc1 out of the nucleus found that this protein and its partner Cdk8 initially left the nucleus for this subnuclear compartment. Only Cnc1 went on to leave the nucleolus and reach the cytoplasm and mitochondria. Control of Cnc1 subcellular trafficking is unexpectedly complex and much is left to understand of this process but this regulatory step requires the presence of both Ste11 and Bck1 to be fully efficient. Data have already been obtained concerning the phosphorylation status of Slt2 in response to Bck1 activation but no information is available concerning the biochemical changes triggered by Ste11. Both the sensors for Ste11 activation and the details of the communication between Rho1 and this MAPKKK remain to be determined.

A differential cellular response to varying degrees of oxidative stress was previously described in the fission yeast *Schizosaccharomyces pombe*. In this yeast, low oxidant treatment requires the action of the basic region-leucine zipper transcription factor Pap1 (a homologue of Yap1 in *S. cerevisiae*) (reviewed in 9). However, when cells are challenged with a severe oxidative stress, Pap1 is inactivated and a new stress response pathway engaged. This pathway is analogous to the CWI pathway and defined by its MAPK Sty1. Sty1 activates function of a new transcription factor designed Atf1 that mounts a transcriptional response to this high oxidant exposure. Atf1 controls expression of genes that permit reduction of the more highly oxidized cysteines that cannot be dealt with by Pap1-dependent genes.

This *S. pombe* system invokes a sequential signaling pathway to deal with the increased oxidative stress while the *S. cerevisiae* signaling to Cnc1 operates through the same pathway. An important future goal for Cnc1 signaling will be to compare the downstream outputs of this pathway that are triggered under conditions of low and high oxidative stress. These data continue to expose the importance of Cnc1 as a critical determinant linking nuclear gene expression profiles to cytoplasmic responses.
